# Advanced Hydrogels With Nanoparticle Inclusion for Cartilage Tissue Engineering

**DOI:** 10.3389/fbioe.2022.951513

**Published:** 2022-06-29

**Authors:** Yunong Ao, En Zhang, Yangxi Liu, Liu Yang, Jun Li, Fuyou Wang

**Affiliations:** ^1^ Center for Joint Surgery, Southwest Hospital, Third Military Medical University (Army Medical University), Chongqing, China; ^2^ Chongqing Institute for Food and Drug Control, Chongqing, China; ^3^ Institute of Life Sciences, Chongqing Medical University, Chongqing, China

**Keywords:** nanoparticles, composite hydrogels, tissue engineering, cartilage, repairing

## Abstract

Cartilage dysfunctions caused by congenital disease, trauma and osteoarthritis are still a serious threat to joint activity and quality of life, potentially leading to disability. The relatively well-established tissue engineering technology based on hydrogel is a promising strategy for cartilage defect repairing. However, several unmet challenges remain to be resolved before its wide application and clinical translation, such as weak mechanical property and compromised bioactivity. The development of nanomedicine has brought a new dawn to cartilage tissue engineering, and composite hydrogel containing nanoparticles can substantially mimic natural cartilage components with good histocompatibility, demonstrating unique biological effects. In this review, we summarize the different advanced nanoparticle hydrogels currently adopted in cartilage tissue engineering. In addition, we also discuss the various application scenarios including injection and fabrication strategies of nanocomposite hydrogel in the field of cartilage repair. Finally, the future application prospects and challenges of nanocomposite hydrogel are also highlighted.

## Introduction

Cartilage injury is a common orthopedic disease with pathogenic factors including sport trauma, arthritis and tumor resection, which mainly presents with joint pain, swelling deformity and dysfunction, seriously affecting the quality of patients’ life (Ao et al., 2019). Articular cartilage belongs to hyaline cartilage, and is mainly composed of water, chondrocytes, type II collagen, and proteoglycans (Tang et al., 2016). Due to the lack of blood vessels, nerves, and lymph, cartilage has limited self-repair capacity after damage, and effective self-healing is not possible for large lesions, therefore the cartilage defect repair attracts a lot of attention in the field of orthopedics. Currently, the treatment for cartilage defects includes arthroscopic debridement, microfracture, cartilage transplantation, and chondrocyte transplantation (Advincula et al., 2021). However, there is still a mismatch between the structure and bio-function of the newly formed cartilage by these strategies and hyaline cartilage, as well as the mechanical properties (Zhang et al., 2016). In addition, for cartilage and chondrocyte transplantation, complications such as possibly harmful effects to the donor sites also limit their application. In the effects to repair cartilage defects effectively and safely, tissue engineering is today an extensively explored strategy due to its unlimited source and tunable physicochemical properties, and a variety of scaffold materials expand more possibilities (Yang et al., 2017).

Hydrogel is one of the promising materials for repairing cartilage due to the easy synthesis and bio-function, which can better simulate the microenvironment of hyaline cartilage, facilitating the proliferation and differentiation of progenitor cells (Miramini et al., 2020; Wang G et al., 2022). However, conventional hydrogel materials often have deficient physicochemical and biological performance in supporting cartilage regeneration. Therefore, an increasing number of studies have managed to combine nanoparticles with biomaterials to form nanocomposite hydrogel in order to improve their mechanical strength, stability and bio-functions (Farokhi et al., 2020; Kashte et al., 2021). In addition, the inclusion of nanoparticles can effectively promote cell attachment, stimulate cell growth and guide tissue regeneration (Li et al., 2016; Armiento et al., 2018). Because nanocomposites can facilitate hydrogels with enhanced mechanical properties, superior stability, and can be synergized with a variety of nanoparticles (NPs) with different preferred functions, they have received much attention since first reported in 2002 when Haraguchi and others introduced exfoliated clay into poly (N-isopropylacrylamide) to form a unique organic/inorganic network with elevated biomechanical properties (Haraguchi and Takehisa, 2002; Merino et al., 2015; Zhang et al., 2016; Zhao et al., 2020). The crosslinked structure between hydrogel polymer and nanoparticles is due to the unsaturated bonds or functional groups on the surface of nanoparticles, which form chemical crosslinking points to enhance the strength of materials and the nanoparticles perform their intrinsic bio-functions *in situ* or after release (Asadi et al., 2018; Niemczyk-Soczynska et al., 2019).

In this review, we focus on the recent research progress of nanocomposite hydrogels in the application of cartilage tissue engineering, summarize the different nanoparticles applied to composite hydrogels and corresponding fabrication strategies in order to promote the wide application of nanocomposite hydrogels in cartilage regeneration.

## Nanoparticles Applied in Cartilage Regeneration

The innovative combination of NPs and hydrogels equips a variety of potential properties to hydrogels that were not present in conventional hydrogels, enhancing the cartilage repair capacity (Eftekhari et al., 2020; Keshvardoostchokami et al., 2021). Nanocomposite hydrogels with various structures and biological functions can be prepared by using different nanoparticles and cross-linking methods, which can be mainly divided into four categories according to their physical and chemical properties: metal and metal-oxide NPs, inorganic NPs, carbon-based NPs, and polymeric NPs (Thoniyot et al., 2015; Liu et al., 2021; Shi et al., 2021). The properties conferring to the composites depend on the incorporated nanoparticle types, such as enhancing cross-linking, improving mechanical strength and promoting cell proliferation. Different kind of nanoparticle hydrogel composites and their associated properties and applications are described below.

### Metal and Metal-Oxide Nanoparticles

In previous studies, metal and its oxides have shown many unique physical and chemical properties, such as the surface plasmon resonance of gold nanoparticles and the broad-spectrum antibacterial properties of silver nanoparticles, and these characteristics can bring more great properties to nanocomposite hydrogels and expand their scope of application (Qiao et al., 2022). For instance, silver nanoparticles (Ag-NPs) have superior antibacterial properties and are widely used in anti-infection and implant surface coating (Barani et al., 2012). Ag-NPs are incorporated into traditional hydrogels to impart conductivity, which affected the swelling rate and conductivity of the hydrogels. The Ag concentration has a direct effect on the conductivity and swelling ratio, with higher concentrations of Ag ions bringing better conductivity, while also reducing the swelling rate and vice versa. Introduced Au nanorods-NIPAAm composite hydrogels and found that if the temperature was higher than the lower critical solution temperature of the gel matrix, the gel structure would collapse, resulting in on-demand burst release of the drug rather than diffusion-controlled release, which could be used in hydrogel drug delivery systems Shiotani et al. (2007). Cu has been studied as an inexpensive antibacterial agent to add to hydrogels, and Cometa and others fabricated an effective Cu-NPs poly (ethylene-glycol-diacrylate) hydrogel antibacterial coating, in which the high surface/volume ratio of Cu-NPs and charged quaternary ammonium salts conferred effective bactericidal capacity to nanocomposite hydrogels Cometa et al. (2013).

In addition to the many applications of metal nanoparticles in hydrogel fabrication, nanocomposite hydrogels prepared with metal oxides are also widely investigated in cartilage tissue engineering. Prepared nanocomposite hydrogels *via* mixing TiO_2_ nanoparticles with chitosan, and the density, compressive resistance, and strength of the composite hydrogels were significantly improved compared with chitosan hydrogel scaffolds without TiO_2_ Kumar (2018). In addition, their research also demonstrated that the addition of TiO_2_ nanoparticles significantly delayed the degradation of hydrogel scaffolds, which can be beneficial for the balance of neo-cartilage formation and scaffold degradation in cartilage tissue engineering. Fe_3_O_4_ can promote the proliferation and chondrogenic differentiation of stem cells, and its nanocomposite hydrogel has been widely investigated to prepare porous scaffolds for cartilage repair. The inclusion of Fe_3_O_4_ in the hydrogel also provides a new strategy for *in situ* monitoring the degradation of the hydrogel scaffold. Combined pulsed electromagnetic fields with Fe_3_O_4_ embedded magnetic nanocomposite hydrogel, demonstrating that the nano hydrogel has good biocompatibility and pulsed electromagnetic field could effectively promote chondrogenic differentiation of seeded bone marrow stem cells (BMSCs) as well as the repair capacity of cartilage defects Huang et al. (2020). When combining ultra-small superparamagnetic iron oxide and cellulose nanocrystal/silk fibroin (SF), also showcased the possibility to monitor hydrogel degradation and cartilage regeneration process in rabbits Chen et al. (2018). Designed injectable dopamine-modified hydrogel microspheres with charge-guided composite inclusion, wherein the modified surface helped the adhesion of spheres to cartilage surface and positively charged drug penetrated the cartilage matrix to target chondrocytes under the guidance of negatively charged cartilage matrix Lin et al. (2021). An efficient drug delivery system for cartilage adhesion, cartilage matrix penetration, and chondrocyte targeting was established, which solved the problem that drugs are difficult to penetrate cartilage matrix, significantly improved the utilization rate and efficacy, and effectively inhibited chondrocyte apoptosis. Metal-charged nanocomposite hydrogels showed good potential for medical applications for the regeneration of cartilage.

Although metal and metal-oxide NPs loaded hydrogels in cartilage regeneration have demonstrated promising results, however to date, there is no well-established preparation protocol or clinical practice to achieve these results, therefore, further comprehensive research is needed. Furthermore, the *in vivo* absorption and metabolism of introduced NPs through hydrogel implantation remains unexplored.

### Inorganic Nanoparticles

In nanocomposite hydrogels, inorganic nanoparticles also present a wide variety of beneficial functions, such as increasing hydrogel hardness and maintaining a good porous structure of the hydrogel. At present, inorganic nanomaterials commonly used in the biomedical field mainly include silica, calcium phosphate and hydroxyapatite nanoparticles.

**FIGURE 1:**
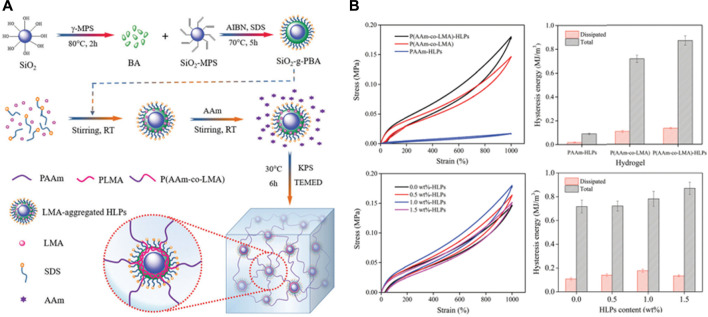
**(A)** Schematic representation of nanocomposite hydrogels reinforced by core-shell SiO_2_-g-PBA hybrid latex particles; **(B)** Loading–unloading cycle behaviors of SiO_2_ incorporated nanocomposite hydrogels. Reproduced from Xia et al. (2017) with permission from Copyright 2017 Royal Society of Chemistry.

Si-NPs were often used as catalysts in previous studies, however the recent reports revealed their unique functions in nanocomposite hydrogel fabrication. Incorporated Si-NPs as cross-linkers into poly (acrylic acid) to fabricate nanocomposite gels, thus generating mechanical properties enhanced composite hydrogels Yang et al. (2013). Si-NPs composite hydrogels have also been shown to have a more sustained drug release profile when loaded with drugs such as doxorubicin. Alvarez and others prepared gentamicin-loaded Si-NPs hydrogel and found that the generated hydrogel could prolong the antibacterial effect through sustained gentamicin release from hydrogel Alvarez et al. (2014). In addition to Si-NPs, SiO_2_ nanoparticles are also widely investigated in tissue engineering because of their large surface area and bio-functions. Reported that the composite hydrogel prepared with SiO_2_ nano-emulsion particles can withstand larger external forces due to the rigid core of SiO_2_, leading to enhanced mechanical properties (Figure 1) Xia et al. (2017). Besides, by incorporating mesoporous silica to modify gellan gum and Manuka honey mixed hydrogel, demonstrated the improved mechanical properties, superior cytocompatibility and antibacterial properties *in vitro* and *in vivo* Bonifacio et al. (2020). Furthermore, silica nanoparticles are also considered as great materials for toughening. Due to their advantages of high modulus, large specific surface area, and diverse functionalization as cross-linking agents in combination with hydrogels, silica nanoparticles have great potential in cartilage repair.

**FIGURE 2:**
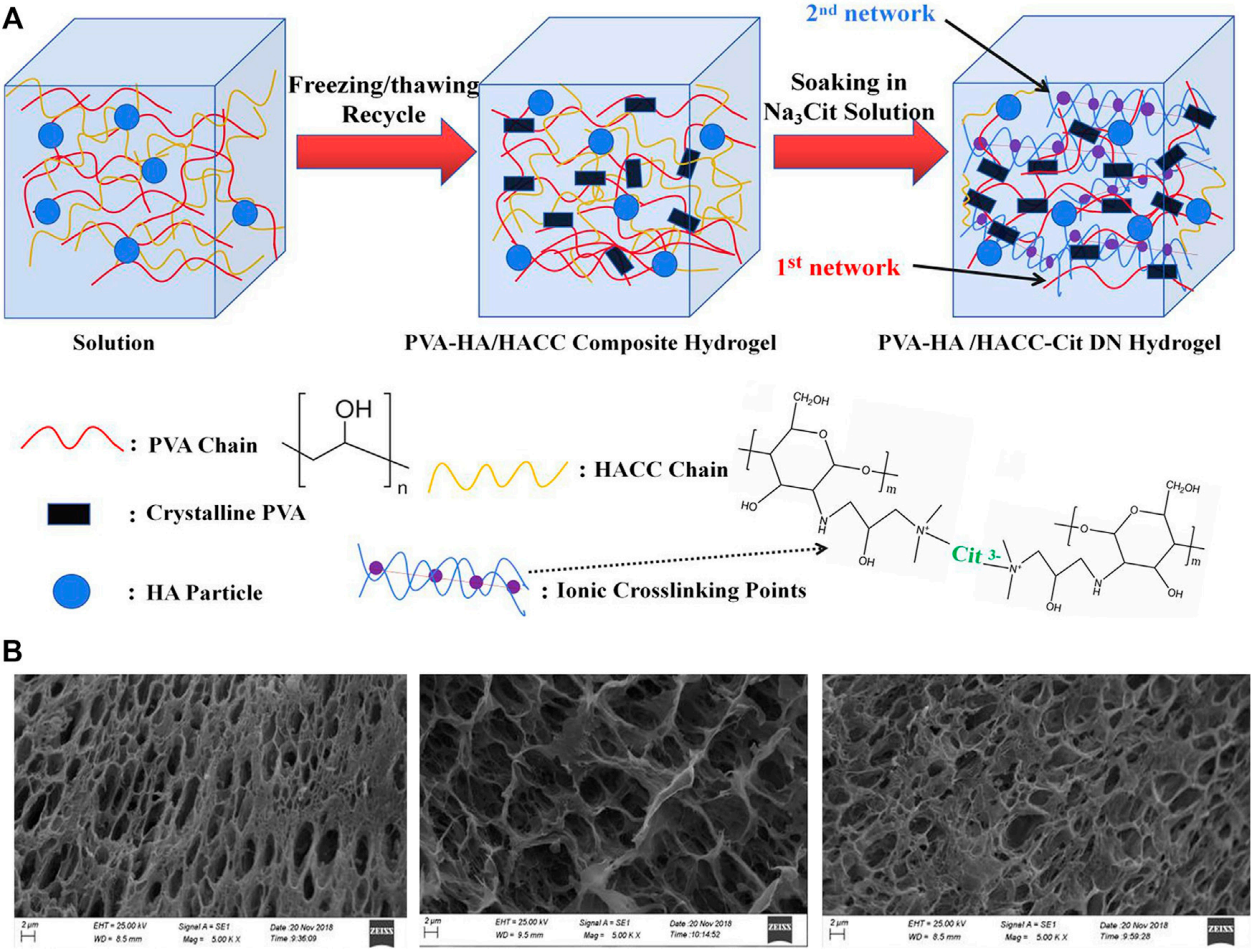
**(A)** Preparation schematic diagram of the preparation of PVA-HA/HACC-Cit DN hydrogelr; **(B)** SEM images of the cross-section of composite hydrogels. Reproduced from Gan et al. (2020) with permission from Copyright 2020 Elsevier.

Nano-hydroxyapatite (nHA) is one of the most used nanomaterials currently, which has the advantages of good biological activity, great cytocompatibility and appropriate degradation ability, can enhance the formation of extracellular matrix mainly type I collagen in the calcified cartilage layer and subchondral bone area by inducing BMSCs to differentiate into osteoblasts, accelerating the formation of calcified cartilage layer and subchondral bone. At the same time, the degradation process of nHA does not produce harmful substances. Proposed composite hydrogels with nHA for the repair of cartilage defects in rabbits, and they found that composite hydrogels had elevated mechanical properties, and were conducive to the adhesion and proliferation of seeded rabbit chondrocytes, with promising potential for the repair of cartilage defects Su et al. (2019). Zhu and coworkers adopted nHA and polylactic acid to prepare cartilage scaffolds and used BMSCs as progenitor cells to repair rabbit full-thickness cartilage defects, the histological and immunohistochemical analyses showed that the cartilage defect was effectively repaired with a large amount of extracellular matrix produced Zhu et al. (2017). A similar repair capacity was also found by through developing composite hydrogels with nHA, adipose-derived stem cells and Kartogenin (KGN) in the rabbit cartilage defect model, and adipose-derived stem cells could maintain good biological morphology and proliferation ability in the composite hydrogels, supporting defect repair Wang Z et al. (2022). Fabricated double-grid nanocomposite hydrogels enhanced by nHA, which showed desirable fatigue resistance and consumption ability because of their unique double-physical cross-linked structure, and the presence of nano-hydroxyapatite helped the nanocomposite hydrogels achieve low friction coefficient, wear resistance, and cytocompatibility (Figure 2) Gan et al. (2020). Produced nanocomposite hydrogels by cryogel method with nHA and chitosan, and found that these nanocomposite hydrogels had interconnected porous structures with water content up to 92%, necessary for cell proliferation and differentiation, can be potentially used for cartilage defect repair Kaviani et al. (2019).

In order to achieve optimal mechanical and biological performance for cartilage repair hydrogels, different inorganic nanomaterials in nature have been investigated, focusing on the optimization of moduli, cytocompatibility and bio-functions. However, it is worth noting that the developed nanocomposite hydrogels are still far away from the natural cartilage in terms of physicochemical and structural geometry. How to adopt different organic hydrogel materials and inorganic nanoparticles to properly simulate the cartilage structure has emerged as a new challenge for tissue engineering. Moreover, there is a paucity of studies reporting the *in vivo* performance of newly developed “optimal” hydrogels based on different inorganic NPs, and further evaluation is required (Sultan and Mathew, 2019).

### Carbon-Based Nanoparticles

Carbon-based nanomaterials applied in tissue engineering mainly include graphene, carbon nanomaterials, carbonized polymers, and carbon dots (CDs), which play a vital role in enhancing physical and chemical crosslinking in carbon-based nanocomposite hydrogels (Wang et al., 2019). CDs have many advantages including low photobleaching, red fluorescence, good uniformity and biocompatibility, which may be useful to study the degradation of cartilage hydrogels *in vivo* or track the release and distribution of drugs (Xia C et al., 2018).

In addition to dispersing stress, articular cartilage also has the function of joint surface lubrication *in vivo*. The nanocomposite hydrogel prepared with CDs can be adopted to enhance the lubricity of tissue-engineered cartilage constructs. Applied carbon nanoparticles to prepare composite hydrogels, and CDs were tightly cross-linked to polymers through stable hydrogen bonds in the hydrogels, with stable physicochemical structures and improved lubricating properties Lu et al. (2018). The results showed that the storage modulus and loss modulus of the composite hydrogel were enhanced by the incorporation of CDs, and the nanocomposite hydrogel has a better shear force and higher viscosity, indicating its potential application for injection or bioprinting. In addition, the swelling rate and tensile strength of the composite hydrogel prepared by CDs were also significantly improved in comparison to those of hydrogel prepared conventionally, which was in line with the needs of cartilage tissue engineering. Carbon-based nanomaterials not only have the advantage of optimizing the physical and chemical properties of hydrogels, but also can stimulate cell proliferation, which is beneficial for the repair of cartilage defects. Demonstrated that multi-walled carbon nanotubes can concentrate more proteins and induce the expression of core-binding factor alpha-1 (CBFA1) and collagen type I alpha-1 (COLIA1), leading to promoted chondrogenic differentiation of human adipose-derived stem cells *in vitro*, this suggests their ability to regulate stem cell responses without exogenous growth factors Li et al. (2020). They also found that the incorporation of carbon nanofibers could improve mechanical and electrical properties, promoting cell proliferation and adhesion ability *in vitro*.

Graphene oxide (GO) is a derivative of graphene, which has many oxygen-containing functional groups on its surface that can be chemically modified (Gopinathan et al., 2017). Introduced graphene oxide into bioink that prepared by photo-crosslinked alginate with gelatin and chondroitin sulfate hydrogel, thereby improving biocompatibility and processability, and this nanocomposite hydrogel simulated cartilage extracellular matrix production and greatly improved the shape fidelity and resolution of the 3D printed scaffold Olate-Moya et al. (2020). The *in vitro* proliferation assay of adipose-derived stem cells showed that the nanocomposite hydrogel scaffold promoted seeded cell proliferation than pure alginate, and could induce cartilage differentiation in the absence of exogenous chondrogenic differentiation factors, which makes it very likely to be used in cartilage tissue engineering.

### Polymeric Nanoparticles

Polymeric nanomaterials are widely used in cartilage tissue engineering mainly including dendrimers, core-shell particles, and liposomes etc. Because polymeric nanomaterials can be cross-linked by stable bonding, resulting in better regularity and mechanical strength, which have attracted special attention (Mohabatpour et al., 2016). At the beginning of this century, research on polymeric nanomaterials in the field of cartilage repair has been conducted. Dendrimers are hyperbranched polymers with external active functional groups that can combine with biomolecules to improve the solubility while effectively loading the drug into the core structure. Söntjens and others synthesized nano hydrogel scaffolds containing biological dendritic polymers for cartilage tissue repair wherein the hydrogel was a triblock copolymer prepared with PEG as the skeleton and dendritic poly (glycerol-succinic acid) as the terminal group Söntjens et al. (2006). The results illustrated that the addition of an appropriate concentration of dendritic polymers was beneficial to the maintenance of round morphology of encapsulated chondrocytes, and promoted the production of type II collagen and proteoglycans. Since then, they had synthesized several hydrogel materials for cartilage repair using PEG as the core and selectively combined with different dendritic macromolecules containing carbamates and ester bonds as terminal groups, illustrating that the compressive stiffness and viscoelasticity of carbamate-crosslinked dendritic polymer-based hydrogels can be comparable to natural articular cartilage through preparation adjusting. And this hydrogel can be injected into cartilage defect in the form of macromolecular monomer solution, followed by photo-crosslinking and can fit into the defect easily.

The spotlight of the dendritic polymer nanoparticles is that different functional groups can be selected and their concentrations are tailorable to match the desired physicochemical properties. Developed a polyamide-amine dendritic polymer (PAMAM) hydrogel to simulate extracellular matrix (ECM) Wang et al. (2014). Among them, the introduction of PAMAM is beneficial to increasing the crosslinking degree, limiting the expansion of hydrogels and improving their mechanical properties, in addition, the increased terminal groups can potentially be used to conjugate more functional groups. The fabricated scaffold promoted the proliferation and differentiation of mesenchymal stem cells without detectable cytotoxicity, which can be potentially applied in cartilage tissue engineering. In the research of Shen and others, they synthesized dopamine modified alginate (Alg-DA) by modifying alginate (Alg) with dopamine (DA), and polydopamine nanoparticles (PDA NPs) were introduced to generate hydrogel scaffold (Alg-DA/PDA). This nanocomposite hydrogel has high porosity, improved mechanical properties, desirable biocompatibility and appropriate degradation ratio. In addition, a novel nanocomposite hydrogel based on gelatin/polycaprolactone-polyethylene glycol-polycaprolactone (Gel/PCEC) was introduced in Asadi’s group by incorporating PCL-PEG copolymer nanoparticles and transforming growth factor-β1 into gelatin hydrogel Asadi et al. (2019). The introduction of PCEC nanoparticles reduced the pore size of the hydrogel and improved the mechanical properties. The fabricated scaffold had good biocompatibility and cell adhesion, and the expression of cartilage-specific ECM genes such as type II collagen and aggrecan were effectively promoted by the designed scaffold. Developed an injectable 3D alginate saline gel including PCL-PEG-PCL (PCEC) microspheres as a carrier for calcium gluconate Liao et al. (2017). The released calcium gluconate promoted the conversion of chondrocyte/alginate suspension and porous microspheres into gels, effectively mimicking the structure of cartilage. The results demonstrated that the nanocomposite hydrogels have properties preferred for cartilage regeneration including pore connectivity, high compressive modulus, good formability and degradation ratio, which can be used as a suitable matrix for cartilage tissue engineering.

Newly developed nanocomposite hydrogels represent a novel class of biomaterials that have the potential to enhance either mechanical or biological performance. While literature indicates an optimal mechanical property, *in vitro* and limited *in vivo* investigations are part of the dogma in the development of nanocomposite hydrogels and mechanisms for the physicochemical and biological properties promotion are not fully investigated systematically. Challenges lie in how to translate their unique properties into clinical applications. In any case, comprehensive *in vitro, in vivo* and long-term clinical characterization of NP biomaterials and implants are necessary.

## Fabrication Strategy

Nanoparticles significantly improve the properties of nanocomposite hydrogels, and their fabrication methods also profoundly affect the physicochemical properties of nanocomposite hydrogel scaffolds. Different fabrication strategy can meet various application requirements, and the main preparation methods of nanocomposite hydrogels include direct injection, electrospinning and 3D printing. In the fabrication process of nanocomposite hydrogels, according to the different timing of the introduction of nanoparticles, it can be mainly attributed to two categories. In the first one, nanoparticles and hydrogels form precast gels and further crosslinking, direct injection and 3D printing fall into this category. In another one, after the crosslinking of hydrogel, the nanoparticles are introduced in the hydrogel matrix, electrospinning falls into this category. The practical application of the different preparation methods and their properties are presented below.

### Directing Injection

Injectable hydrogels allow *in situ* repair of cartilage defects by invasive techniques, wherein progenitor cells and growth factors can be delivered through hydrogel to enhance the clinical efficiency (Li et al., 2022; Luo et al., 2022). In comparison to rigid scaffold implantation, hydrogel injection has more advantages in tissue engineering due to its flexible traits in shape adaptability, which is more potential in clinical applications (Taymouri et al., 2021). In recent years, a large number of studies have been conducted to develop nanocomposite hydrogels for direct injection. For example, Obtained injectable hydrogel using polyethylene glycol-poly [L-alanine-poly (L-aspartic acid)] (PEG-PA-PD) triblock copolymers and layered double hydroxide (LDHs) nanocomposites, and performed *in vitro* experiments with tonsil-derived mesenchymal stem cells Lee et al. (2017). Compared with the traditional hydrogel system, the nanocomposite system was helpful to improve cell aggregation and significantly increased the expression of chondrogenic differentiation markers. In addition, the surface of LDHs can be modified by interaction with a variety of other bioactive molecules to design and prepare a variety of injectable nanocomposite hydrogels that can be used for cartilage repair.

**FIGURE 3:**
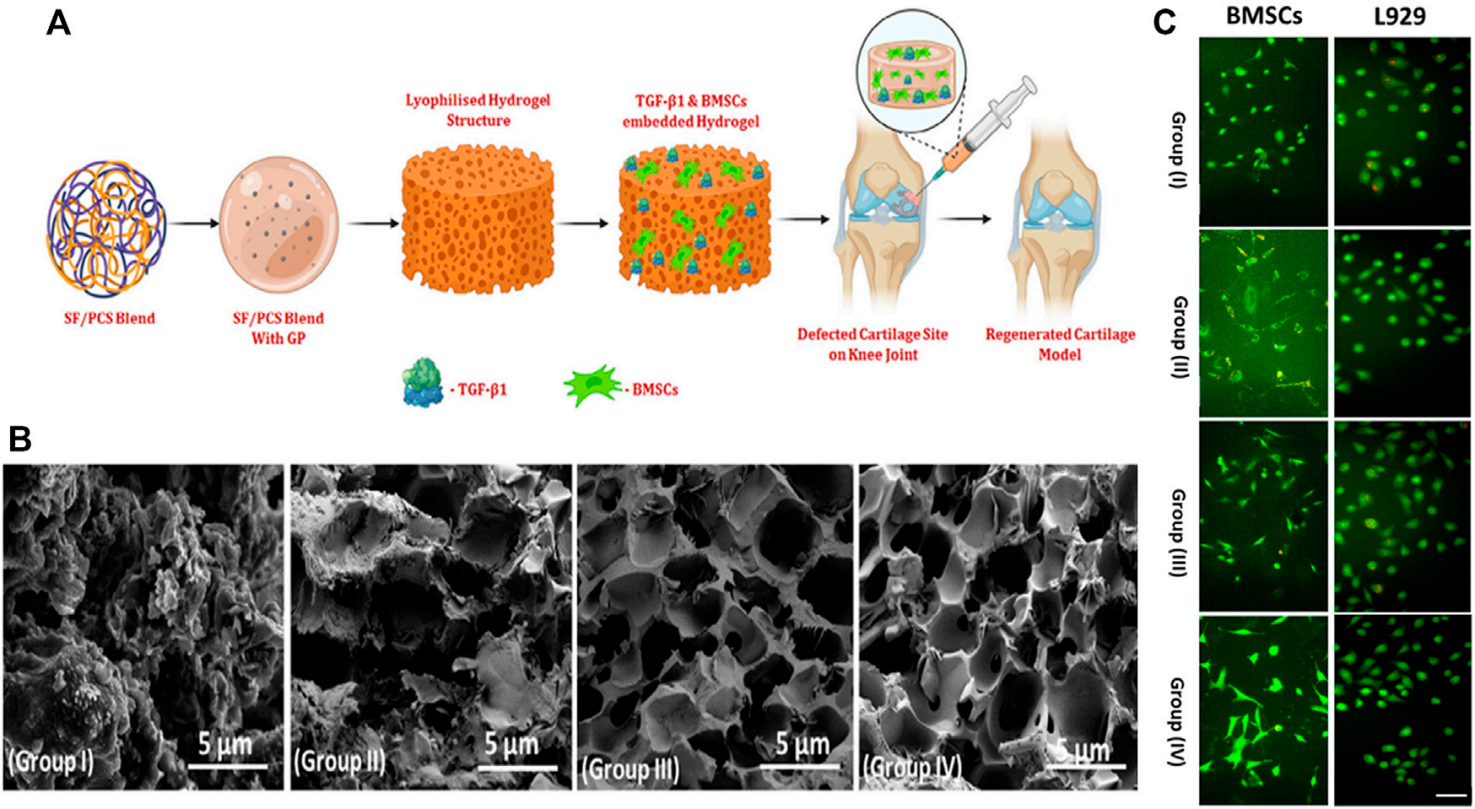
**(A)** Schematic illustration of BMSCs and TGF-β1 encapsulated SF/PCS injectable hydrogel for cartilage repair; **(B)** The morphological of prepared hydrogel groups with different blending ratios were observed and visualized by SEM analysis; **(C)** Fluorescence microscopic observation of BMSCs and L929 seeded on different hydrogels after 24 h incubation. Reproduced from Zheng et al. (2021) with permission from Copyright 2022 Wiley.

However, in order to realize the clinical application of injectable hydrogels, it is necessary to fabricate hydrogel with similar biomechanical properties and composition to human cartilage. Generated silk fibroin-based injectable nanocomposite hydrogels, which have great gel morphology and porosity, and can improve mechanical properties, cell proliferation and nutrient exchange in cell-based therapy (Figure 3) Zheng et al. (2021). The results of the co-culture of injectable nano hydrogel with human fibroblasts and BMSCs showed that it had good biocompatibility. Boyer and coworkers prepared a nano-reinforcement clay reinforced hydrogel with an interpenetrating network, and verified that the injectable nanocomposite hydrogel can promote the secretion of glycosaminoglycans and enhance mechanical properties Boyer et al. (2018). Injectable hydrogels are an effective option that can be applied for cartilage repair, as also confirmed by results from animal experiments. Introduced an injectable hydrogel with Kartogenin-encapsulated nanoparticles for repairing porcine cartilage defects Yan et al. (2020). The results of macroscopic observation, micro-computed tomography and histologic revealed that this injectable nanocomposite hydrogel facilitated the repair of hyaline cartilage and subchondral bone in the porcine model. As a convenient and minimally invasive surgical approach, injectable nanohydrogels have important clinical application prospects.Electrospinning

Electrospinning is the preparation of one-dimensional nanoscale fibers by spraying polymer solutions, main electrospinning equipment is composed of a high-voltage power supply device, metal needle syringe and grounding device (Zhang et al., 2020). The preparation technique applied to electrospun nanofibers is relatively simple, and this method can deal with various natural materials or synthetic polymers. Choosing appropriate materials and parameters can easily control the properties of the prepared fibers and apply them in a variety of fields. Electrospun nanofibers have the potential for tissue engineering due to their high surface-to-volume ratio and high porosity (Song et al., 2021). The unique fibrous structure is able to withstand the stress experienced in various tissues, which is very much in line with the needs of cartilage scaffolds. For example, prepared PCL-based composite nanofiber scaffolds by incorporating different concentrations of GO and surface grafted PEG-modified GO-g-PEG through electrospinning, which have high mechanical strength and superior cytocompatibility and can be further used for cartilage repair Scaffaro et al. (2017). In order to obtain a three-dimensional porous nanofiber-reinforced hydrogel scaffold mimicking cartilage extracellular matrix, Gunes and others produced poly (3-hydroxybutyrate-co-3-hydroxyvalerate) nanofiber reinforced carboxymethyl chitosan-silk fibroin hydrogel *via* wet-electrospun Gunes et al. (2020). This composite scaffold obtained by dispersing wet electrospun nanofibers in a polymer matrix can maintain a stable interconnected microporous structure, which supported the differentiation of bone marrow stem cells. In the research of Hejazi’s team, they fabricated three-dimensional nanofibrous scaffolds similar to the native cartilage structure, assuming that cells can grow under specific conditions and repair damaged tissue Hejazi et al. (2021). In this study, the gradient nanofiber scaffold was mainly composed of five layers and different materials were adopted according to unique tissue structures, including PCL, gelatin, nHA and chitosan. The multilayered scaffold structure exhibited better mechanical properties and promotes cell proliferation and has great potential in the treatment of cartilage defects. Generated nanocomposite hydrogels using highly negatively charged star-shaped poly (ethylene glycol)/heparin hydrogels (sPEG/Hep) as cartilage extracellular matrices *via* electrospinning technique, and the hydrogels combined with mPCL melt electrospun fiber networks showed mechanical morphology and internal structure similar to native cartilage, providing a suitable microenvironment for chondrocyte culture *in vitro* Bas et al. (2017). Subsequently, they also used finite element to build models to further simulate the deformation mechanism of the hydrogel structure and to predict the compressive modulus.

**FIGURE 4:**
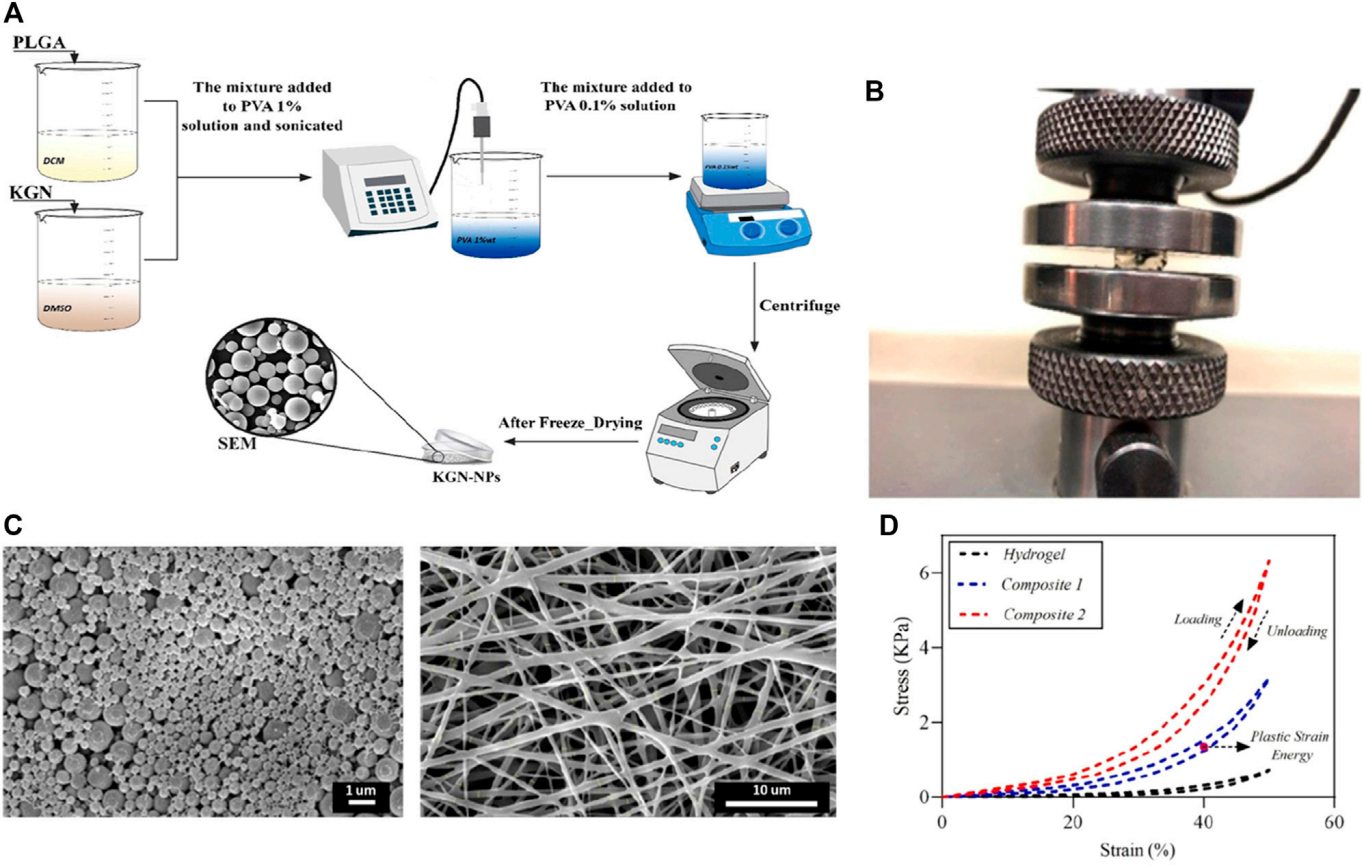
**(A)** Schematic illustration of the synthesis of KGN-PLGA nanoparticles; **(B)** The mechanical test of fabricated samples; **(C)** The FESEM images of KGN-PLGA nanoparticles and Gelatin electrospun mat; **(D)** The results of hysteresis loop at 50% strain. Reproduced from Zare et al. (2021) with permission from Copyright 2021 Elsevier.

Electrospinning technology is also applied to drug sustained release and delivery systems because of its unique preparation technology, and the characteristics are also useful for the treatment of cartilage diseases. Applied coaxial electrospinning technology to encapsulate glucosamine sulfate (GAS) into the core of polycaprolactone (PCL) nanofibers, and this nanofiber structure can allow the continuous release of GAS over time. The results confirm that coaxial electrospinning can prepare GAS-loaded nanofibers with good tensile properties (Chen et al., 2020). *In vitro* experiments have shown that this nanofiber has a significant effect on the proliferation of rat articular chondrocytes, demonstrating its promise application in the field of cartilage tissue engineering. In another study, Zare and coworkers applied electrospinning to prepare composites synthesized from alginate, Kartogenin, and PLGA (Figure 4) Zare et al. (2021). As a scaffold/drug delivery system, the elastic modulus of this composite is significantly increased than that of the traditional hydrogels, and the results of resazurin test and Live/Dead staining confirmed that the composite can promote adipocyte mesenchymal stem cells to maintain better cell morphology and viability.

### 3D Printing

**FIGURE 5:**
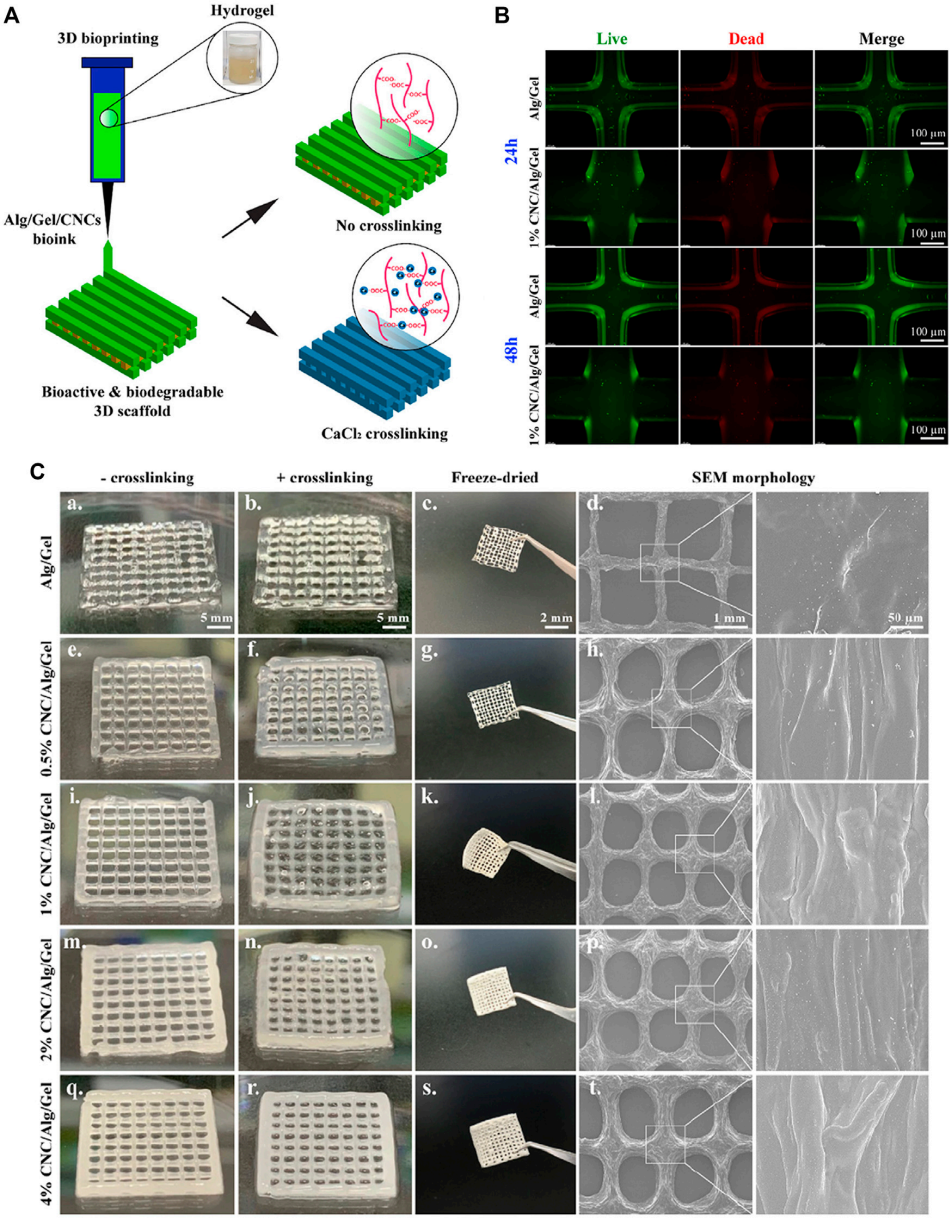
**(A)** Schematic representation of 3D bioprinting using Alg/Gel/CNCs hydrogels for tissue engineering; **(B)** Images of Live/Dead assay of 3D bio-printed cell-laden constructs (Alg/Gel and 1% Alg/gel) at indicated time intervals; **(C)** Digital photographs showing the un-crosslinked, crosslinked, and fridge-dried scaffolds after 3D printing; SEM images showing the internal structure of printed scaffolds. Reproduced from Dutta et al. (2021) with permission from Copyright 2021 Elsevier.

3D printing can fabricate a variety of customizable complex structures and has been widely used in many fields (Li P et al., 2021; Li Q et al., 2021). Among them, the application of 3D printing in the field of tissue engineering can endow the 3D structure fabrication, facilitating the incorporation of target cells and growth factors so as to construct biologically active tissue or organ analogs (Han et al., 2021; Wei et al., 2021). Previous studies have reported the preparation of hydrogel scaffolds *via* 3D printing. For example, used silk fibroin hydrogel modified with glycidyl methacrylate as bioink for 3D printing, and verified that it was a feasible strategy to form silk fibroin hydrogel scaffold with good biocompatibility and complex structures Kim et al. (2018). Introduced nanocomposite hydrogel constructs *via* 3D printing and post-photo-crosslinked, which achieved the regulation of macroscopic shape and internal pore structure, and regenerated mature cartilage with cartilage-specific ECM Xia H et al. (2018). Dutta and others designed 3D-printable hybrid biodegradable hydrogels, which is composed of alginate, gelatin, and cellulose nanocrystals (Figure 5) Dutta et al. (2021). They demonstrated that the 3D printed scaffold can provide a favorable environment for cell proliferation, adhesion and nutrient exchange in tissue engineering applications.

3D printed cartilage scaffold with nanocomposite hydrogel can also promote cartilage repair by controlling the sustained release of incorporated growth factors. Reported two kinds of nanocomposite hydrogels that were prepared by introducing hydrothermally treated nHA and poly (lactic-co-glycolic acid) (PLGA) nanoparticles with nucleocapsid structure Castro et al. (2015). The results illustrated scaffolds fabricated with a composite hydrogel containing nHA were more conducive to the proliferation of MSCs than the PLGA hydrogel, while increased the compressive modulus as well. Nanocomposite hydrogel containing PLGA nanoparticles and TGF-β1 could achieve sustained release of TGF-β1 in fabricated cartilage scaffolds.

The natural cartilage has a gradient structure, and the artificial scaffolds are expected to have similar mechanical properties. There have been a large number of studies on joint cartilage, and 3D printing provides an effective way to better repair cartilage by preparing multilayer hydrogels with gradient hardness (Mancini et al., 2020). For instance, prepared cartilage repair scaffolds containing GO and HA nanoparticles *via* 3D printing. The designed scaffold had a specific structure with a smooth and flat surface, which was conducive to material load-bearing Meng et al. (2020). And the distance between the stents from top to bottom increased layer by layer, which was conducive to the formation of a firm connection between the material and the bone base, while there was no interface for the tight connection between each layer so as to prevent the loosening and shedding of the sample after implantation. In addition, it is concluded that the introduction of GO-HA can reduce the intermolecular hydrogen bonding and molecular entanglement density, and improve the dynamic viscosity of hydrogels. The solution shows obvious shear behavior in the range of printing shear rate, which can effectively avoid extrusion expansion, so improving the applicable range of sample printing and the condition parameters in the printing process. Reported a 3D printed nanocomposite scaffold to mimic the natural structure of artificial cartilage Liu et al. (2020). They firstly prepared PLGA electrospun nanofiber incorporated hydroxybutyl chitosan hydrogels and then injected it into PCL framework with an internal microchannel, which offered the nanocomposite hydrogel with preferable mechanical support and substance exchange. Their research suggested that 3D printing is a feasible strategy to regulate the inner structure for providing an ideal biomimetic microenvironment for chondrogenic differentiation.

The properties of the material itself used for 3D printing also have a large impact on the scaffold performance, for example, prepared cell-free bilayer scaffolds by low-temperature deposition using GelMA as a matrix with HA incorporated into the subchondral scaffold Gao et al. (2021). The effects of line spacing and line width of printed scaffolds on the cartilage regeneration in rabbits were evaluated, including the pore size, porosity, specific surface area and mechanical strength etc. Their results showed that with the increase of line spacing, the mechanical properties of the materials progressively decreased. The properly spaced scaffold presented cartilage regeneration with cartilage lacunae and subchondral bone formation. Therefore, the tractable rheological behavior of soft matter can be used to improve 3D printing, while bilayer hybrid scaffolds generated by continuous 3D printing are expected to be biomaterials for regenerating articular cartilage.

Cartilage tissue engineering aims to generate autologous cartilage-like grafts for defect therapy by defined positioning of growth factors, progenitor cells, and biomaterials in a 3D hierarchical manner, leading to the generation of mature cartilage tissue. Ongoing challenges toward functional cartilage regeneration, such as structural reconstruction, chemical simulation and cellular incorporation, are unlikely to be overcome by a single biomaterial and manufacturing technology. Advanced bio-fabrication strategies, through combining different nanocomposite hydrogels and manufacturing technologies are an emerging trend toward the recapitulation of the delicate and intricate structure of natural cartilage. However, following the current publications, it is still a challenge to speculate on how the optimal cartilage biomaterial/grafts should be developed to maximize regeneration capacity upon injection or implantation. Possibly, the revolution of new strategies that are capable of closely recapitulating the macro-/micro-structure of cartilage tissue and allowing the precise placement of different progenitor cells, growth factors, or bioactive elements will help achieve the desired biological functions.Conclusion and Future Perspectives.

Nanomedicine has been applied in different fields of biomedicine, including diagnosis, imaging, pharmaceutics and regenerative medicine, which also brings new directions for cartilage tissue engineering technology and contributes to cartilage regeneration and repair. Nanocomposite hydrogels change the physical and chemical function as well as biocompatibility of hydrogels based on the unique properties of nanoparticles, and the strong support of scaffold structure avoids the damage of hydrogels under compression *in vivo*, which has a promising application prospect in the field of cartilage tissue engineering. At present, although great progress has been made in the study of cartilage tissue engineering, it still faces many challenges and difficult problems. For example, whether the nanocomposite hydrogel can sustain good physicochemical properties under load-bearing conditions and provide strong support in cartilage defects. Degradability is also an important factor to be considered in the application of nanocomposite hydrogels in cartilage tissue engineering, and the degradation time should neither be too long nor too fast. On the one hand, it needs to provide physical and biochemical microenvironment support during tissue healing, and on the other hand, it also needs to have suitable degradability to reduce the adverse effects caused by the long-term residual of the scaffold material.

In this review, we introduce the relevant research progress of nanohydrogels for cartilage repair, and summarize the main nanomaterials currently applied in the preparation of nanocomposite hydrogels and related preparation techniques. Different fabrication techniques have their specific advantages, for example, 3D bio-printing fabricates hydrogel scaffolds with controllable porosity and internal structure, which is conducive to the transport of nutrients and the discharge of metabolites, and the characteristics can promote the migration, proliferation and differentiation of seeded cells in cartilage tissue engineering, which is of great significance for cartilage defect repair. However, the current materials science and engineering approach typically focus on the material design and scaffold geometry optimization concerning mechanical properties, geometry as well as unique bio-functions by means of *in vitro* testing or small animal evaluation. Many of them are yet to be evaluated by *in vivo* large animal studies and become the convention in clinical practice. Furthermore, *in vitro* and *in vivo* investigations to date typically emphasize biocompatibility and cartilage regeneration performance with a focus on very limited parameters in the short term. There is often little to no investigation in the longer term.

Promising future directions in nanocomposite hydrogel development for cartilage regeneration is the decoupling of different research in material development, scaffold design, fabrication optimization and adaptation of the implant following long-term cartilage remodeling. In the short term, nanocomposite hydrogels do not have subversive therapeutic effects on cartilage repair. There is still a long way to apply nanocomposite hydrogels in the clinical repair of cartilage, and different nanomaterials have their unique characteristics. How to combine these characteristics for cartilage repair in order to obtain the best repair effect remains unexplored.
